# Supplementary figures and images for: The performance of interrupted time series designs with a limited number of time points: Learning losses due to school closures during the COVID-19 pandemic
Source: PLoS One. 2024 Aug 7;19(8):e0301301. doi: 10.1371/journal.pone.0301301 (PMC11305537; doi:10.1371/journal.pone.0301301)

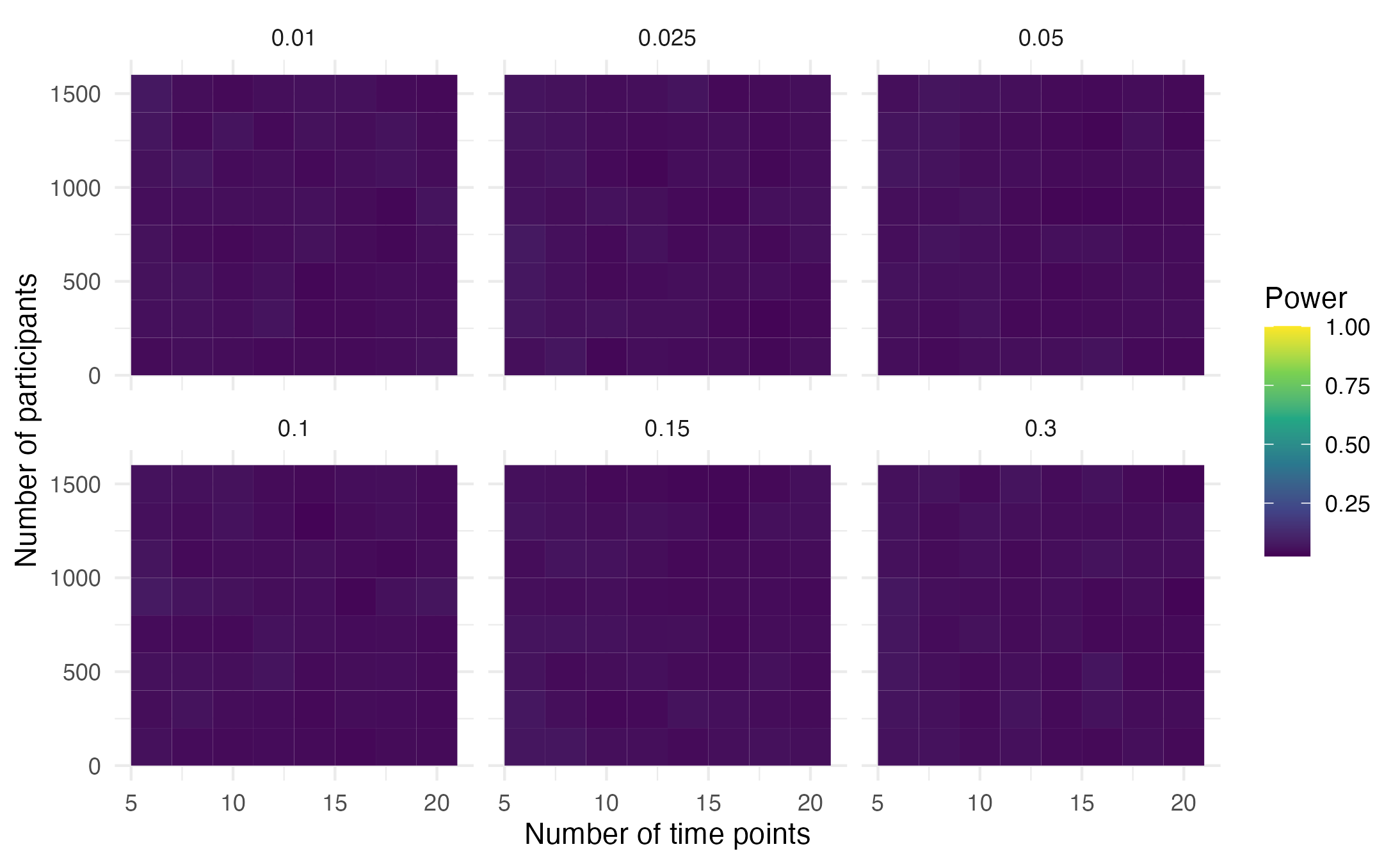

Supplement: S1 Fig — (TIF) [file pone.0301301.s001.tif]

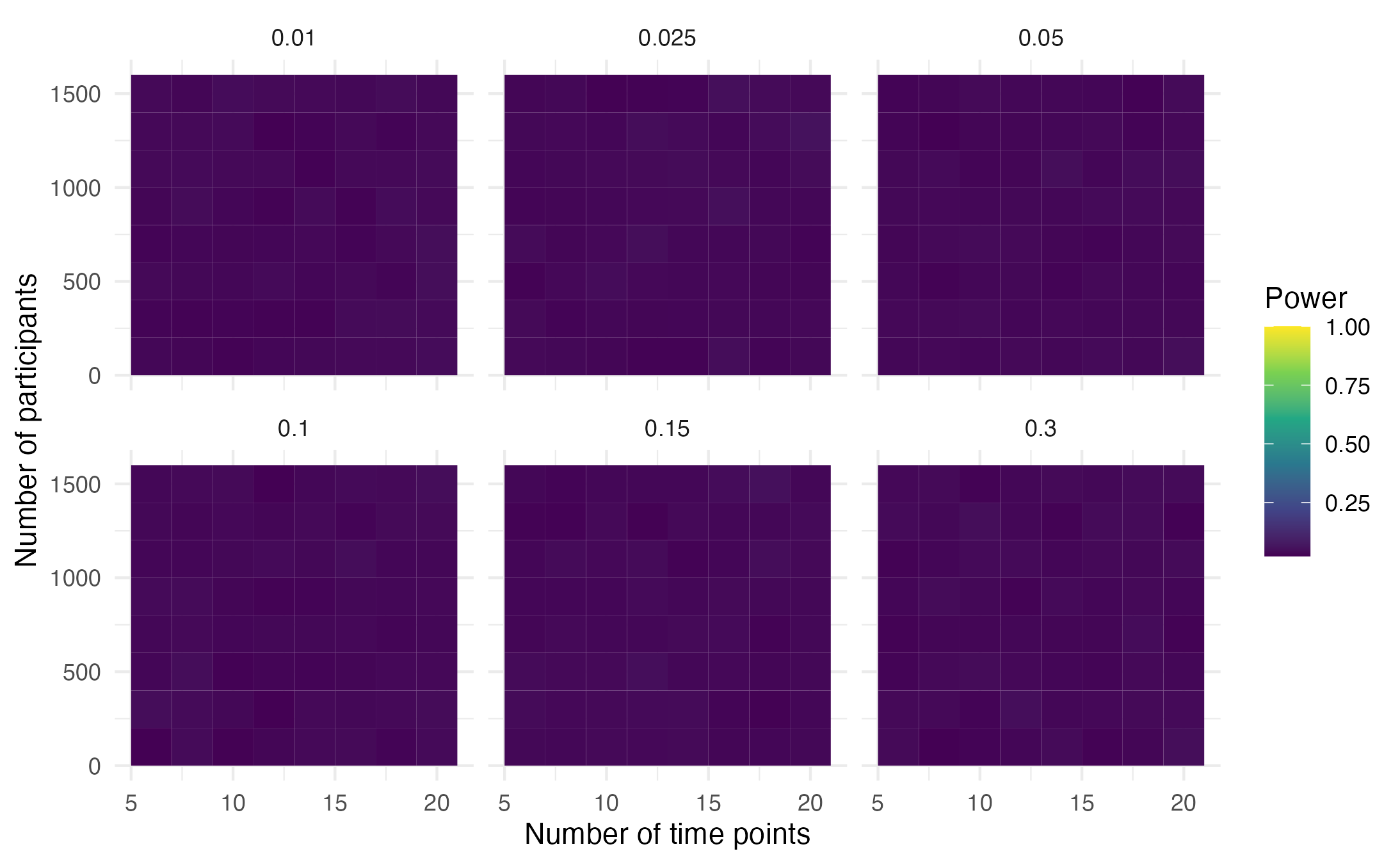

Supplement: S2 Fig — (TIF) [file pone.0301301.s002.tif]

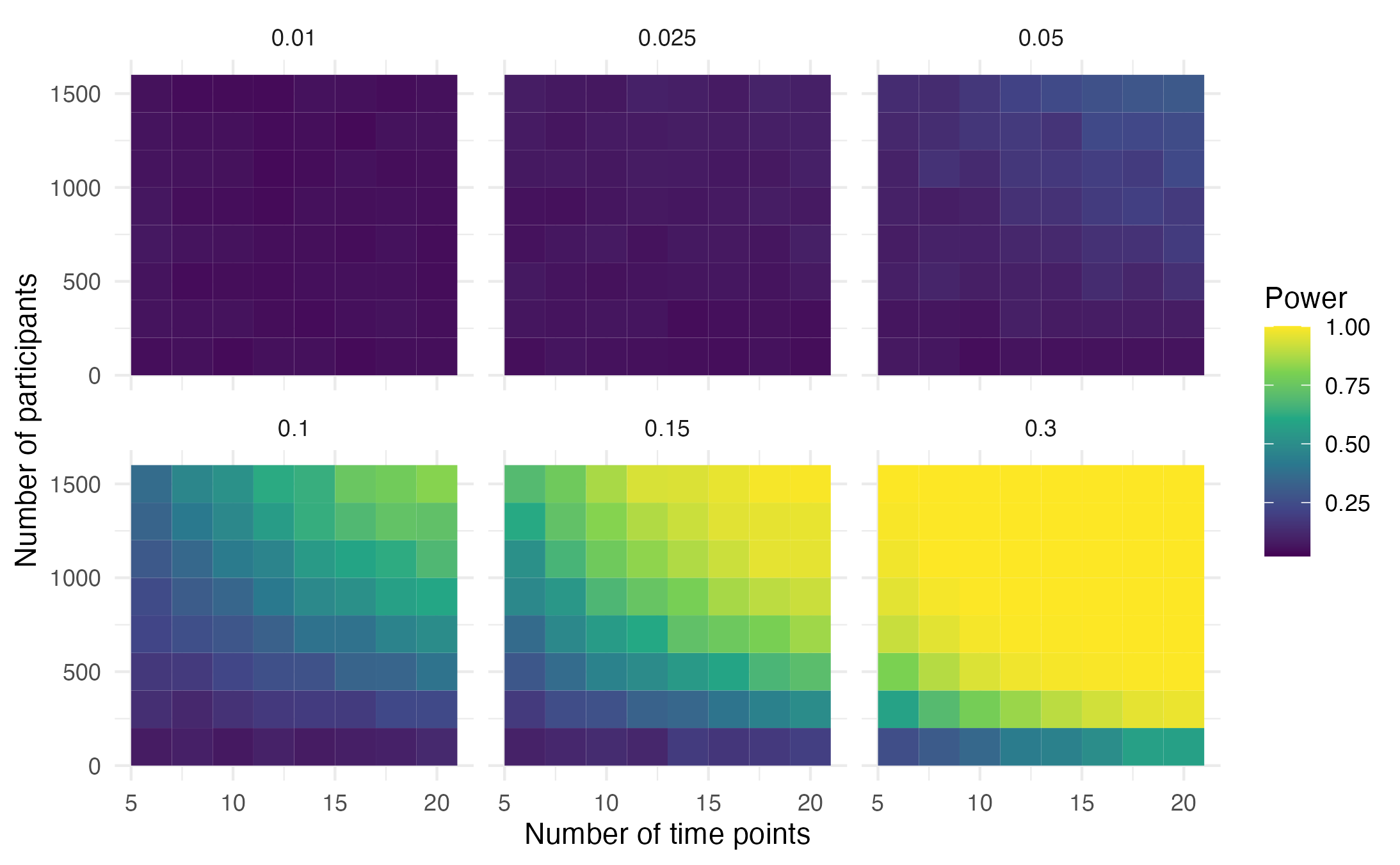

Supplement: S3 Fig — (TIF) [file pone.0301301.s003.tif]

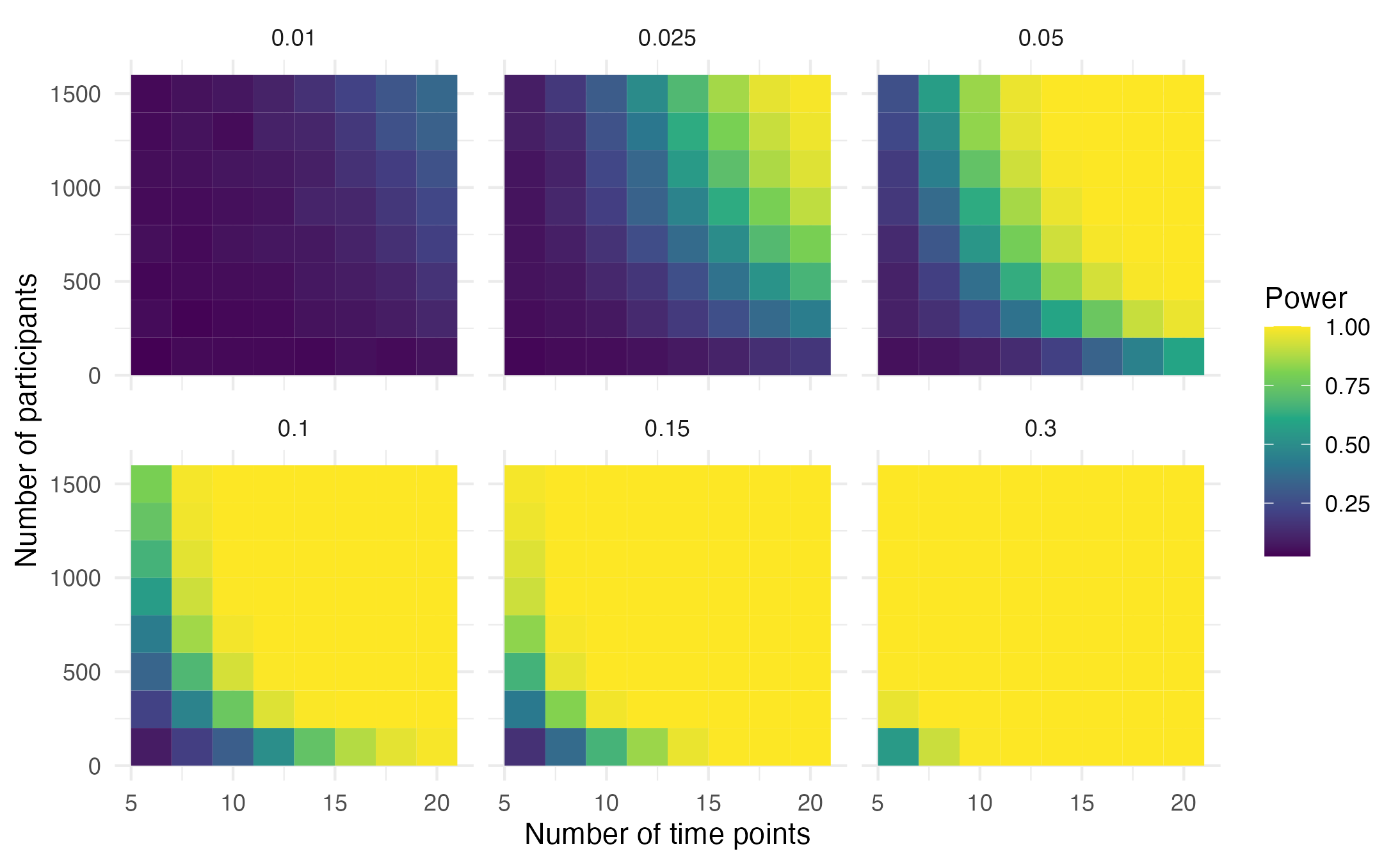

Supplement: S4 Fig — (TIF) [file pone.0301301.s004.tif]

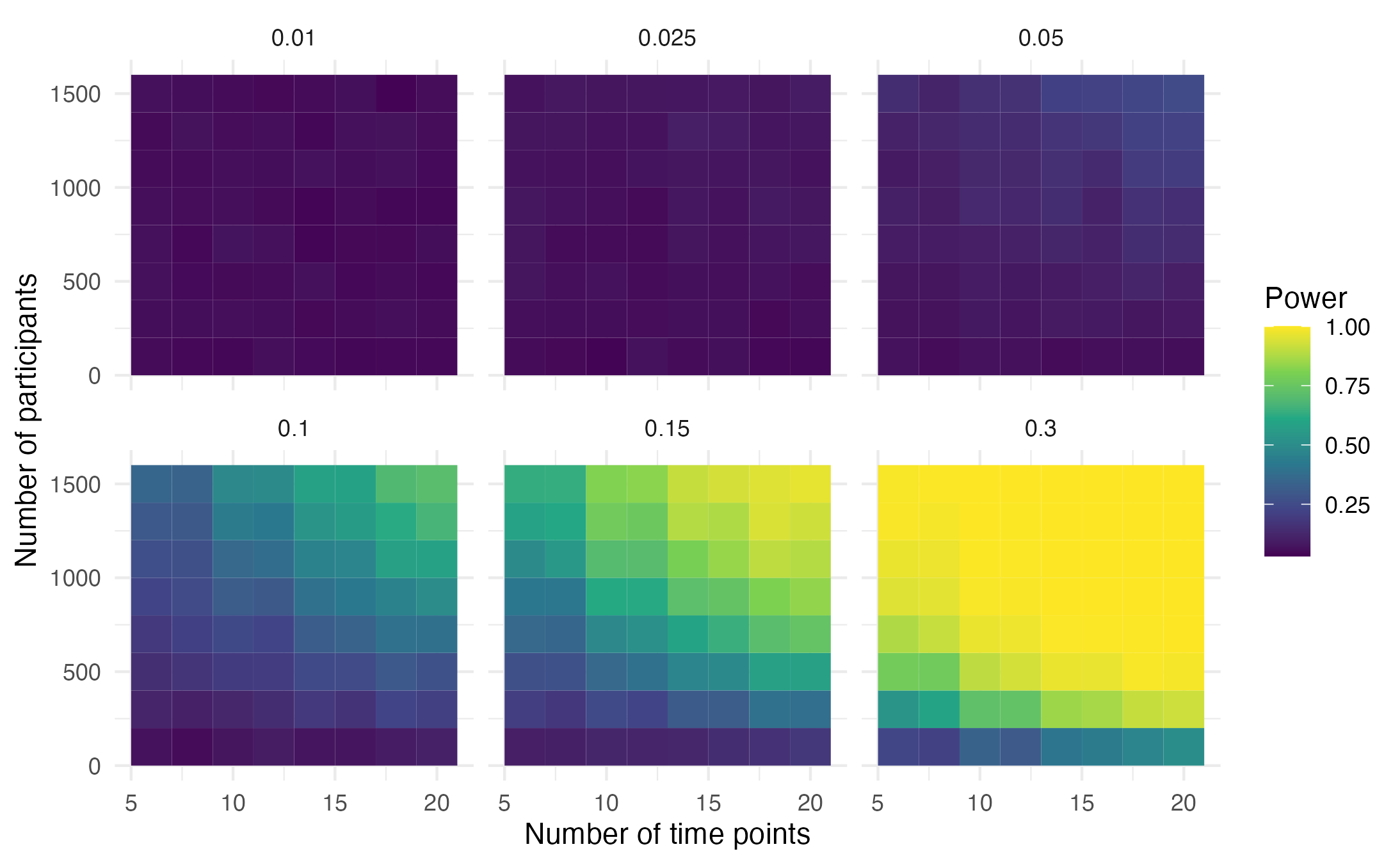

Supplement: S5 Fig — (TIF) [file pone.0301301.s005.tif]

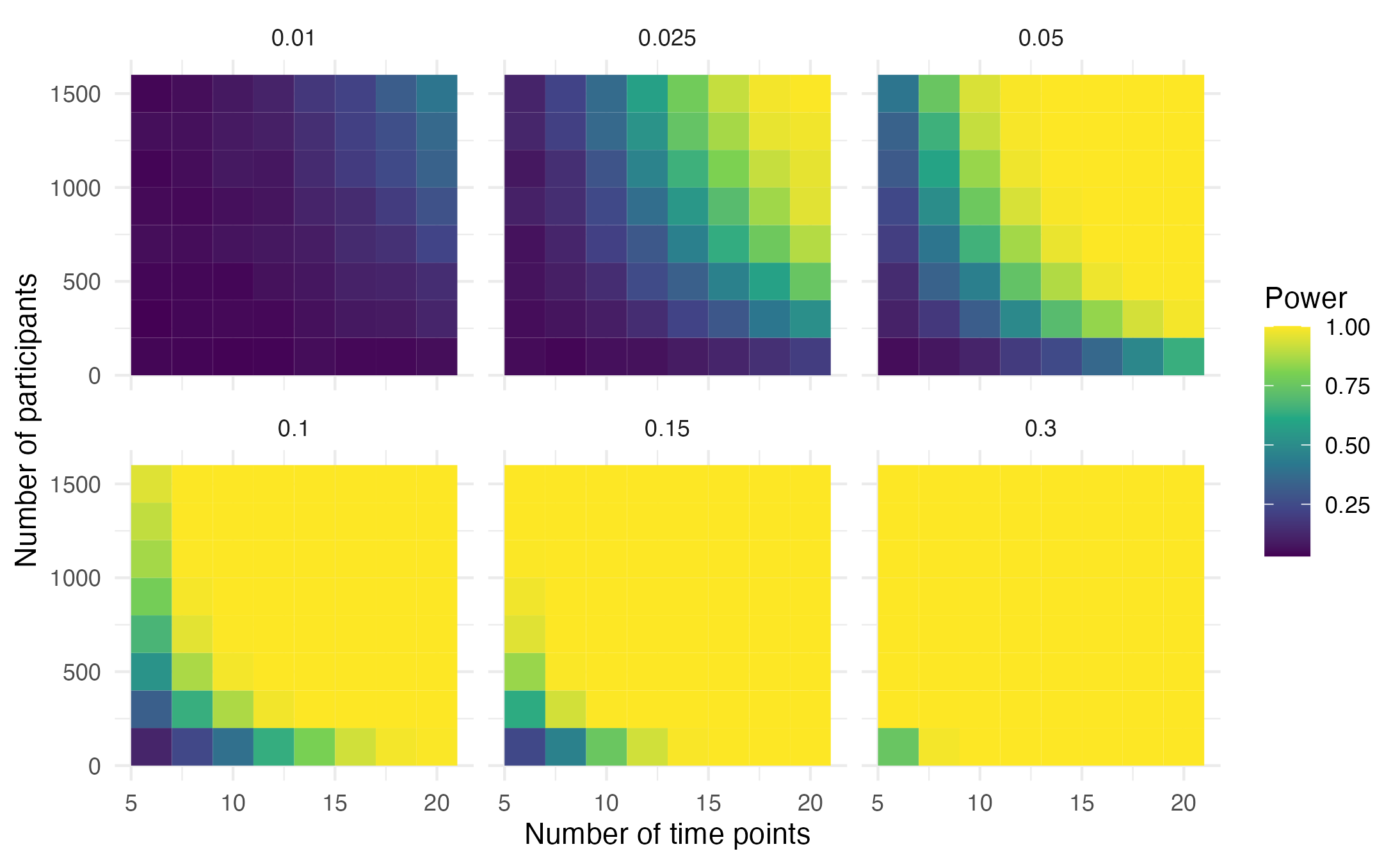

Supplement: S6 Fig — (TIF) [file pone.0301301.s006.tif]

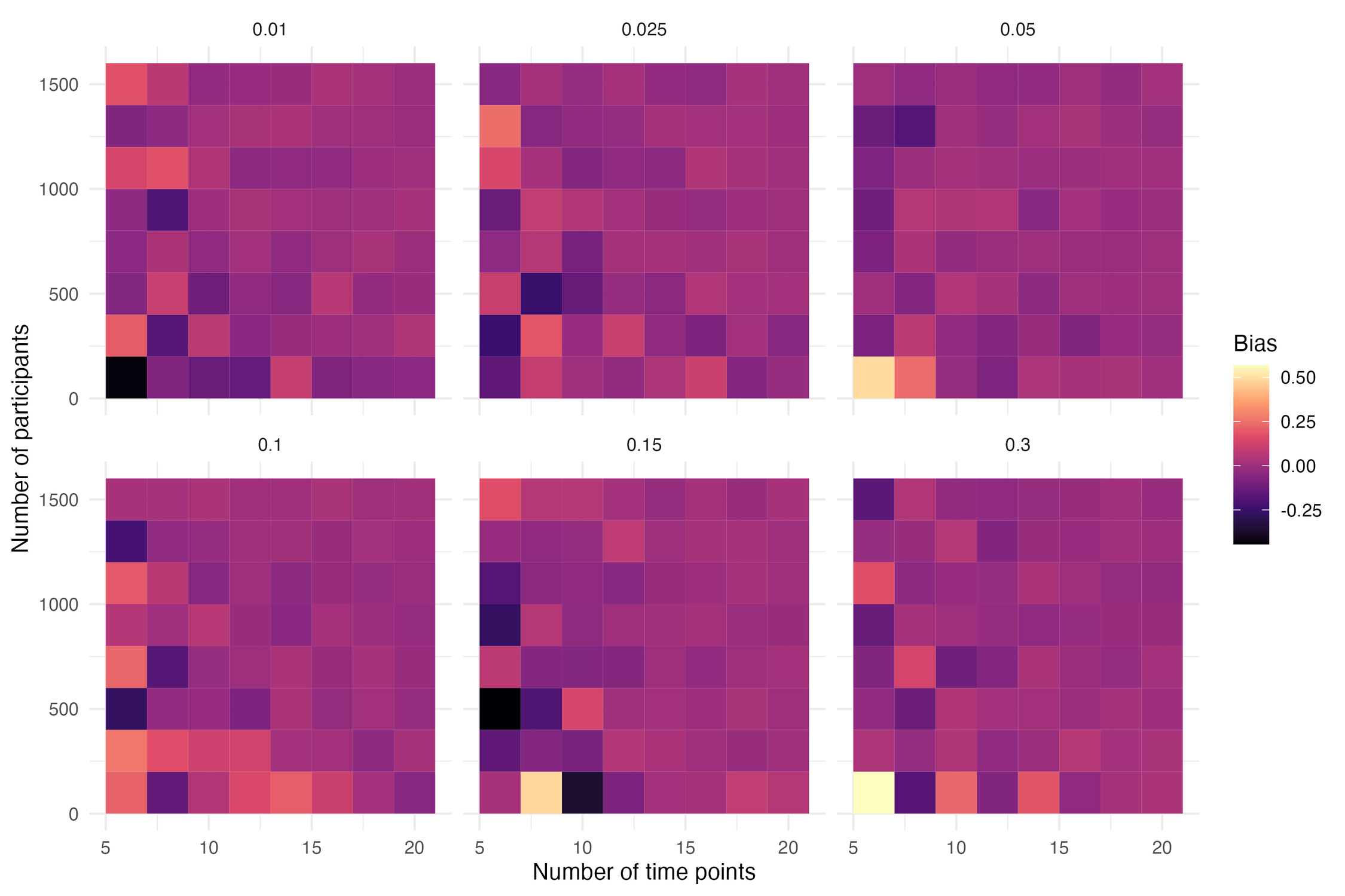

Supplement: S7 Fig — (TIF) [file pone.0301301.s007.tif]

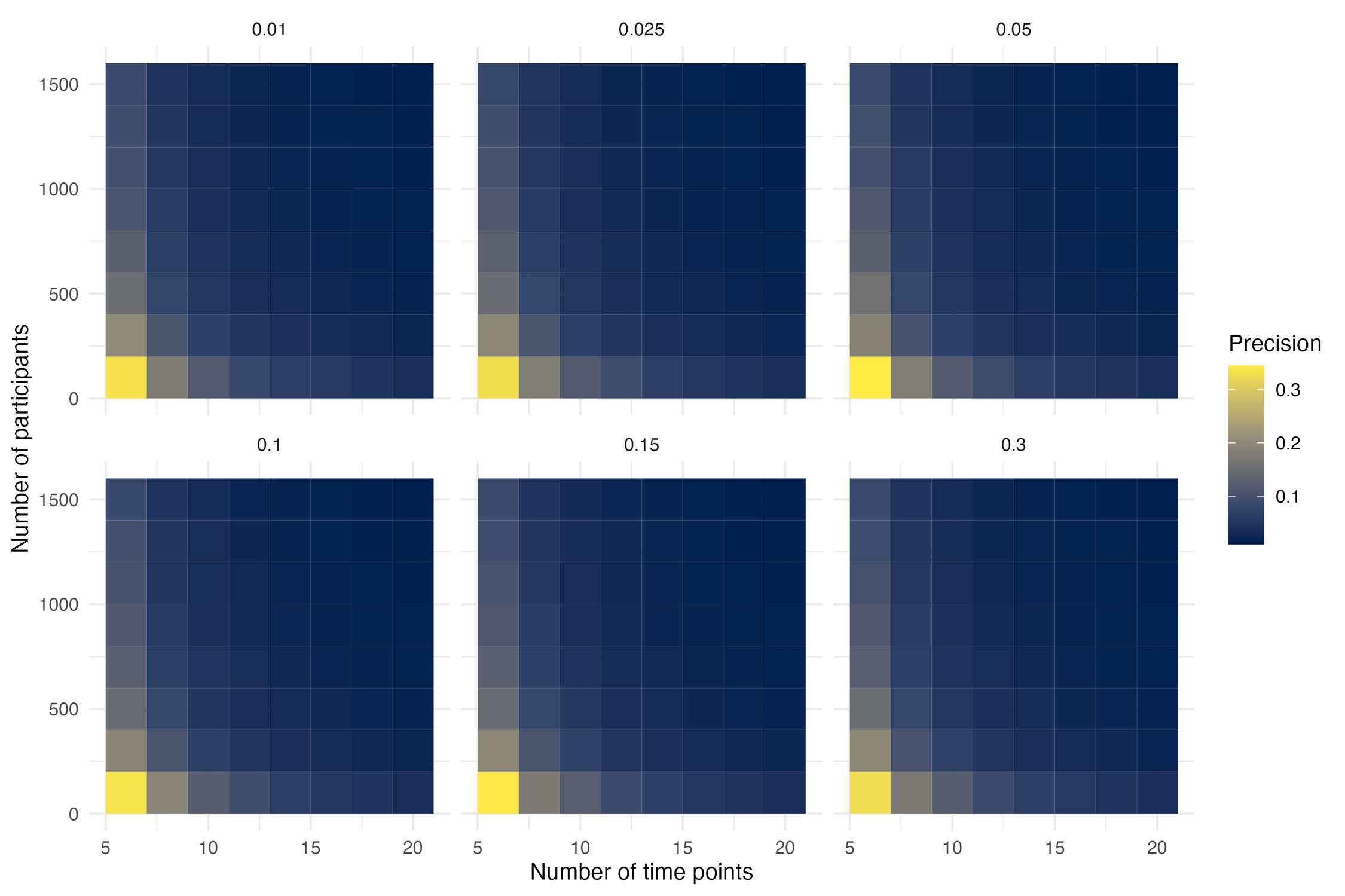

Supplement: S8 Fig — (TIF) [file pone.0301301.s008.tif]
