# Supplementary material for: The performance of interrupted time series designs with a limited number of time points: Learning losses due to school closures during the COVID-19 pandemic
Source: PLoS One. 2024 Aug 7;19(8):e0301301. doi: 10.1371/journal.pone.0301301 (PMC11305537; doi:10.1371/journal.pone.0301301)
Supplement: Attachment — Submitted filename: ID PONE-D-23-39424 Reviewer.doc [file pone.0301301.s009.doc]

**Comment on Manuscript**

Manuscript Number: **PONE-D-23-39424**

Full Title: **The Performance of Interrupted Time Series Designs with a Limited Number of Time Points: Learning Losses due to School Closures during the COVID Pandemic**

*Submitted to* ***Plos One*** *(SCIE, IF = 3.72)*

The interrupted time series (ITS) have been addressed in this study. The Monte Carlo simulation method has been selected to serve the proposed issues. Some results collected have been presented in this study. This study is interesting however there are some drawbacks that the authors should address them to improve this study.

1. There are a lot of typing mistakes which have been found in this paper.
2. The main contribution of this study must be provided.
3. The proposed models must be indicated clearly.
4. The evaluation and analysis of collected results must be indicated clearly.
5. The significance of this study must be provided in the conclusion part.
