# Supplementary material for: The performance of interrupted time series designs with a limited number of time points: Learning losses due to school closures during the COVID-19 pandemic
Source: PLoS One. 2024 Aug 7;19(8):e0301301. doi: 10.1371/journal.pone.0301301 (PMC11305537; doi:10.1371/journal.pone.0301301)
Supplement: Attachment — Submitted filename: Response to Reviewers.docx [file pone.0301301.s010.docx]

**Point-by-point reply letter to reviewer comments of article PONE-D-23-39424**

“The performance of interrupted time series designs with a limited number of time points: Learning losses due to school closures during the COVID-19 pandemic”

We want to thank the (associate) editor(s) and anonymous reviewers for their comments on our earlier version of the manuscript “The performance of interrupted time series designs with a limited number of time points: Learning losses due to school closures during the COVID-19 pandemic”.

In this letter we provide a point-by-point reply to the comments provided, and indicate how we have changed the main text of the paper. The comments have been helpful, especially in putting more emphasis on the main contribution of the paper, and several stylistic and textual changes. We are confident the manuscript has been improved, and are looking forward to see this manuscript published in PLos One.

Comments by the (associate) editor(s):

1. Please ensure that your manuscript meets PLOS ONE's style requirements, including those for file naming. The PLOS ONE style templates can be found at 
[https://journals.plos.org/plosone/s/file?id=wjVg/PLOSOne_formatting_sample_main_body.pdf](https://journals.plos.org/plosone/s/file?id=wjVg/PLOSOne_formatting_sample_main_body.pdf) and 
[https://journals.plos.org/plosone/s/file?id=ba62/PLOSOne_formatting_sample_title_authors_affiliations.pdf](https://journals.plos.org/plosone/s/file?id=ba62/PLOSOne_formatting_sample_title_authors_affiliations.pdf)

2. We suggest you thoroughly copyedit your manuscript for language usage, spelling, and grammar. If you do not know anyone who can help you do this, you may wish to consider employing a professional scientific editing

All authors went through the manuscript again, and have made about 150 changes to the manuscript in terms of spelling, style and grammar. We attach a version of the manuscript with tracked changes, so it is easy to see what we changed. The manuscript has benefited from this.

3. Please provide additional details regarding participant consent. In the ethics statement in the Methods and online submission information, please ensure that you have specified what type you obtained (for instance, written or verbal, and if verbal, how it was documented and witnessed). If your study included minors, state whether you obtained consent from parents or guardians. If the need for consent was waived by the ethics committee, please include this information.

We obtained written consent from the ethical review board. We have added this to the text, and along with the registration number for this study, it is possible to locate our ethical review at Utrecht University.

Once you have amended this/these statement(s) in the Methods section of the manuscript, please add the same text to the “Ethics Statement” field of the submission form (via “Edit Submission”).

We have added this as well.

4. PLOS requires an ORCID iD for the corresponding author in Editorial Manager on papers submitted after December 6th, 2016. Please ensure that you have an ORCID iD and that it is validated in Editorial Manager. To do this, go to ‘Update my Information’ (in the upper left-hand corner of the main menu), and click on the Fetch/Validate link next to the ORCID field. This will take you to the ORCID site and allow you to create a new iD or authenticate a pre-existing iD in Editorial Manager. Please see the following video for instructions on linking an ORCID iD to your Editorial Manager account: [https://www.youtube.com/watch?v=_xcclfuvtxQ](https://www.youtube.com/watch?v=_xcclfuvtxQ)

We have added the ORCID ID “0009-0009-7092-2848” for the corrspondinng author into Editrial Manager.

5. Please ensure that you refer to Figure 11 in your text as, if accepted, production will need this reference to link the reader to the figure.

We have added this reference to Figure 11 to the text.

Comments by Reviewer 1:

This is a nice study examining the potential of ITS on naturalistic settings with the covid-19 pandemic as an example. I would recommend emphasizing the key contribution of this study in more detail in the abstract. It is nice that the authors highlight the potential of the ITS for such settings as other analyses such as the difference-in-difference approach also have their limitations.

The article is overall well-written and I did not detect potential flaws. AS such, I have no more concrete points to add.

We thank the reviewer for these comments. We have rewritten parts of the abstract, introduction, background and discussion sections to put more emphasis on the contribution of this article, and the potential of the ITS model, following similar comments by reviewer#2.

Comments by Reviewer 2:

 The interrupted time series (ITS) have been addressed in this study. The Monte Carlo simulation method has been selected to serve the proposed issues. Some results collected have been presented in this study. This study is interesting however there are some drawbacks that the authors should address them to improve this study.

1.There are a lot of typing mistakes which have been found in this paper.

All authors went through the manuscript again, and have made about 50 changes to the manuscript in terms of spelling, style and grammar. We attach a version of the manuscript with tracked changes, so it is easy to see what we changed. The manuscript has benefited from this.

2.The main contribution of this study must be provided.

Here, we made several changes to the abstract (based on #R1 comments), the introduction, background and discussion to highlight the main contribution (under what circumstances can the ITS be used). The version with tracked changes provides can be used to easily see these changes.

3.The proposed models must be indicated clearly.

Equations 1 to 5 show the basic ITS model, along with several variations, all building on the standard regression model in equation 1, so that it is easy to readers familiar with regression what the ITS model does. In table 5 we have indicated the specific simulation scenario’s and model used to evaluate the ITS. These models in terms of notation directly relate to equations 1 to 5. We hope this clarifies for the reviewer the models we use.

4.The evaluation and analysis of collected results must be indicated clearly.

We did not alter the results section greatly (apart from grammar and spelling), but we did add a “reading guide” at the start of the results to guide readers through this section:

“The performance of the ITS design is discussed per simulation scenario in the order of Table 5.

The most relevant figures for the power, bias and precision are shown in the main text. The performance measures are discussed in the performance section. The remainder of the figures can be found in the appendix. The heatmaps indicate the performance of the models, as can be inferred by the colour, for different scenarios of the number of time points, sample size and effect size. Using heatmaps allows for seeing patterns in the results.”

5.The significance of this study must be provided in the conclusion part.

This comment is related to comment 2. We have changed the last part of this section to highlight the significance of the study:

“In a nutshell, we conclude that the interrupted time series design is a strong method for assessing changes over time, even with few time points. This paper shows that the notion that many time points are always necessary for an ITS analysis is shown to be incorrect. Additionally, this paper shows that employing ITS designs can be difficult when the data are complex, such as multiple interventions or non-linear time effects.

The results from the simulation study can be used as a guide for future research. Researchers should thus inspect their data, hypothesize about effect sizes, and consider multiple models to be used for estimation to decide if there is enough power.”
