# Supplementary material for: The performance of interrupted time series designs with a limited number of time points: Learning losses due to school closures during the COVID-19 pandemic
Source: PLoS One. 2024 Aug 7;19(8):e0301301. doi: 10.1371/journal.pone.0301301 (PMC11305537; doi:10.1371/journal.pone.0301301)
Supplement: Attachment — Submitted filename: ID PONE-D-23-39424-R1 Reviewer.doc [file pone.0301301.s011.doc]

**Comment on Manuscript**

Manuscript Number: **PONE-D-23-39424R1**

Full Title: **The Performance of Interrupted Time Series Designs with a Limited Number of Time Points: Learning Losses Due To School Closures During the COVID-19 Pandemic**

*Submitted to* ***PLOS ONE*** *(SCIE, IF = 3.7)*

The authors have addressed some comments from this reviewer. However, there are some existing points that the authors should address them to improve this study.

1. The keywords must be implemented in this study.
2. The authors must explain why the Monte Carlo simulation method has been employed in this study.
3. The discussion part must be implemented to analyze the prioritization and the comparison with the experimental working from the proposed methods.
4. The conclusion must be re-written shortly to describe the significance of this study.
5. The quality of figures is very poor. Kindly provide the high quality solution of these figures.
