# Supplementary material for: The performance of interrupted time series designs with a limited number of time points: Learning losses due to school closures during the COVID-19 pandemic
Source: PLoS One. 2024 Aug 7;19(8):e0301301. doi: 10.1371/journal.pone.0301301 (PMC11305537; doi:10.1371/journal.pone.0301301)
Supplement: Attachment — Submitted filename: Response to Reviewers.docx [file pone.0301301.s012.docx]

**Reviewer #1:** All comments have been nicely addressed and I have nothing to add. Really nice work that is timely and will be interesting to read for others.

We want to thank the reviewer for reviewing our manuscript again, and are happy we have been able to address all comments

**Reviewer #2:** The authors have addressed some comments from this reviewer. However, there are some existing points that the authors should address them to improve this study.

reply: We have tried to address these remaining concerns by changing the manuscript in some specific sections:

1.The keywords must be implemented in this study.

reply: We have added the following keywords: Natural experiment, Interrupted time series, Segmented regression, Simulation study, Power. All are named numerus times in the manuscript and play a vital role in the article.

2.The authors must explain why the Monte Carlo simulation method has been employed in this study.

reply: We have added a short section in the methods section to explain why we use a Monte Carlo simulation study: "A Monte Carlo simulation study is used to assess the performance of different ITS designs over 384 scenarios. Monte Carlo simulation allows us to estimate our statistics of interest by eliminating the noise caused by random sampling [51]. This allows us to inspect under which circumstances the ITS designs can estimate an effect well. In the simulation study, data are generated based on predefined parameters. These are then compared to the estimates to evaluate the performance of the models."

3.The discussion part must be implemented to analyze the prioritization and the comparison with the experimental working from the proposed methods.

reply: we changed the discussion section in two ways (also to adress the next comment).

1. We added a sentence to the main findings, to illustrate the implications of the simulation: "This research has investigated the performance of the standard ITS design as well as that of various extensions, in situations with limited data. While the ITS design works well under many different circumstances, there are some factors to consider before running an analysis: the sample size in the case of a step change, the number of time points for a slope change, and the problem of underfitting\overfitting. Not one of these factors alone can account for the power, it always depends on the a combination of factors.
2. We have added a conclusion section (see also next comment)

4.The conclusion must be re-written shortly to describe the significance of this study.

reply: we have restructured the discussion section, and now added a separate conclusion section

5.The quality of figures is very poor. Kindly provide the high quality solution of these figures.

reply: the figures are now all available as vector-based figures, so quality issues should no longer be there.
